# Amplicon of 16S rRNA Gene Sequencing of Fertilized Volcanic Soils from Southern Chile

**DOI:** 10.1128/MRA.00590-20

**Published:** 2021-03-25

**Authors:** Rodrigo A. Vargas, Natalia Valdés, Iván Balic, Eduardo I. Contreras, Carlos Venegas, Carlos P. Aranada, Mario Tello, Alex R. Gonzalez

**Affiliations:** a Laboratorio de Microbiología Ambiental y Extremófilos, Departamento de Ciencias Biológicas y Biodiversidad, Universidad de Los Lagos, Osorno, Chile; b Laboratorio de Metagenómica Bacteriana, Facultad de Química y Biología, Universidad de Santiago de Chile, Santiago, Chile; c Departamento de Acuicultura y Recursos Agroalimentarios, Universidad de Los Lagos, Osorno, Chile; d Centro de Educación y Tecnología (CET), Chonchi, Chiloé, Chile; e Departamento de Ciencias Biológicas, Universidad de los Lagos, Osorno, Chile; University of Maryland School of Medicine

## Abstract

The volcanic soils of Chiloé Island, Chile, have physical and chemical characteristics that affect their productivity. We report here a 16S rRNA gene analysis that characterizes the predominant microbial communities in volcanic soils of Chiloé either in the presence or absence of fertilization. The major phyla identified were *Proteobacteria*, *Acidobacteria*, and *Actinobacteria*.

## Abstract (precis)

The volcanic soils of Chiloé Island, have physical and chemical characteristics that affect their productivity. We report here a 16S rRNA gene analysis that characterizes the predominant microbial communities in volcanic soils of Chiloé either in the presence or absence of fertilization. The major phyla identified were *Proteobacteria*, *Acidobacteria*, and *Actinobacteria*.

## ANNOUNCEMENT

Agricultural soils are subject to degradation due to the use of monocultures, excessive tillage, little rotation, and erosion. These practices modify the microbial community, in diversity and abundance, reducing organic matter and wear of macronutrients (1). Organic and inorganic fertilizers have been used to reverse soil degradation, maintaining the productivity of the crops. These fertilizers positively impact the bioavailability of nutrients and the structure and functionality of the soil microbial community (2, 3). Chiloé Island in the south of Chile has a volcanic origin, marked by physicochemical characteristics such as a large amount of organic matter (20% to 35%), high retention rate of N-P, and low mineralization rate (4). However, little information is available about the microbiota that characterizes these soils and how fertilization influences their composition and structure (5).

In order to address this issue, on December 2017, soil samples were collected in an experimental field of Chiloé (42°38′32.9″S, 73°48′08.9″W; Centro de Educación y Tecnología, Comuna de Chonchi, Región de Los Lagos, Chile). Three sample types were included, control soil without any type of fertilization treatment (C), organic soil treated with fermented sheep feces (179 kg ha^−1^) (O), and a soil treated with inorganic fertilizer containing nitrogen (10%), phosphorus pentoxide (20%), and potassium oxide (10%) (200 kg ha^−1^) (I). The samples were taken in triplicate in each of the three plots (2 m^2^). Approximately 200 g was randomly collected in each plot by taking a profile of the first 10 cm of soil with a soil corer. Total DNA was extracted from 0.3 g of soil sample using the DNeasy PowerSoil isolation kit (Qiagen, USA).

The 16S rRNA gene from variable region V4 was amplified using primers 515F and 806R (6) with barcodes on the forward primer in a 28-cycle PCR using the HotStarTaq Plus master mix kit (Qiagen, USA). PCR products were purified using Ampure XP beads and used to prepare an Illumina DNA library with a TruSeq Nano kit. 16S amplicon sequencing was performed at Molecular Research DNA (MR DNA; Shallowater, TX) with the MiSeq reagent kit v3 on the Illumina MiSeq platform (2 × 300-bp paired ends [PE]) following the manufacturer’s guidelines. A total of 566,729 reads with an average size of 250 bp were processed using an MR DNA analysis pipeline that removed barcodes and primers, denoised sequences, and eliminated sequences of <150 bp. Default parameters were used for all software. The operational taxonomic units (OTUs) with a 97% similarity cutoff were then clustered using USEARCH v11 (7) and classified using BLASTN (8) against a database derived from Ribosomal Database Project II release 11 (http://rdp.cme.msu.edu) and NCBI (www.ncbi.nlm.nih.gov) after identification and removal of chimeras using a modified UCHIME v4.1 algorithm (9). The total OTUs in the 9 samples were 17,616. The OTUs corresponding to mitochondria, chloroplasts, and eukaryotes were eliminated at the time of generating the taxonomic assignment graph.

**FIG 1:**
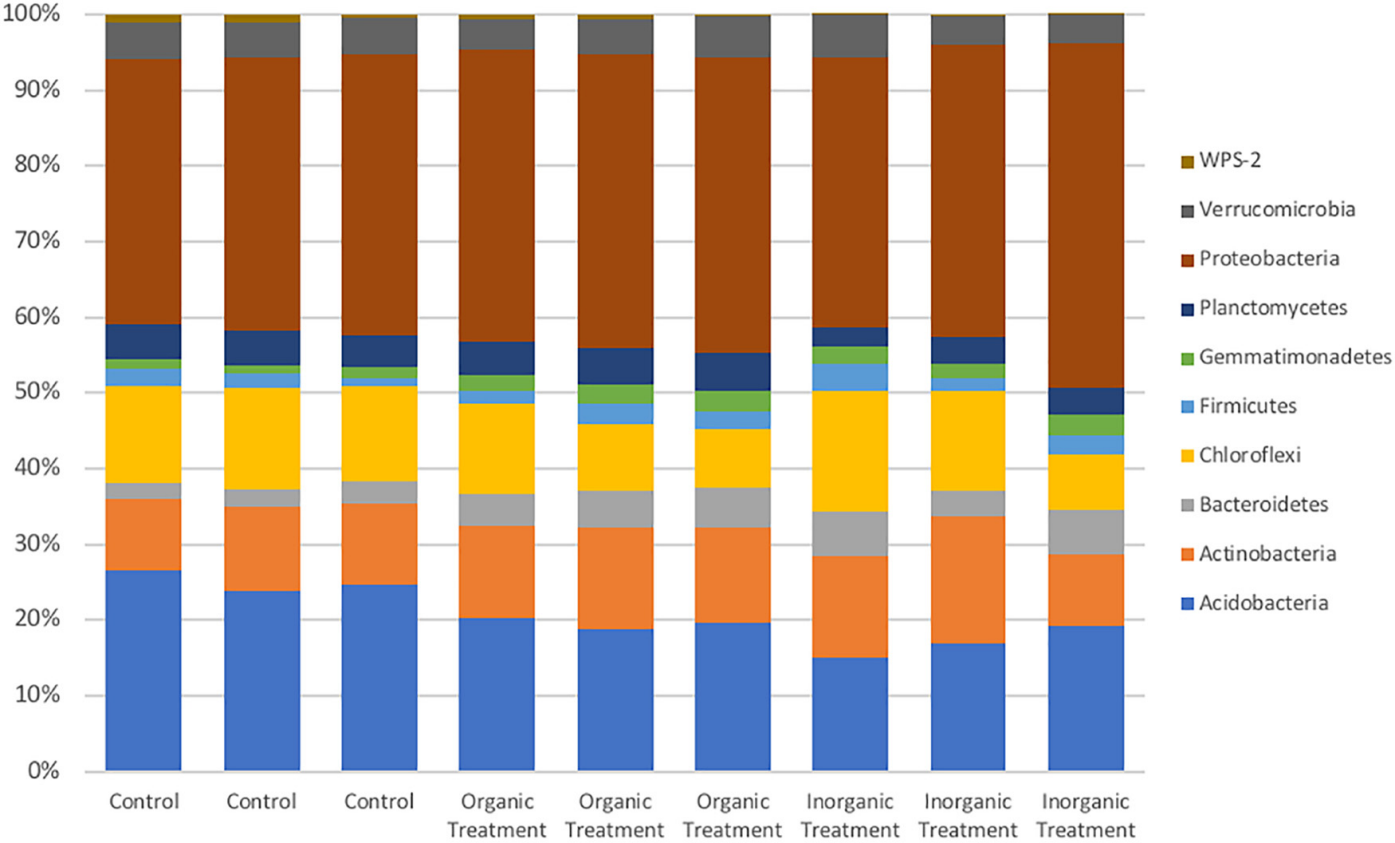
Microbial composition of treated and untreated soils. The figure shows the relative abundance of the main phyla (>1%) identified by 16S rRNA amplicon sequencing in soils untreated (control) and treated with organic or inorganic fertilizers (organic and inorganic treatment, respectively). The sequences without taxonomic assignment were not included.

We identified 10 main phyla (>1% of abundance) present in all soils analyzed (Fig. 1), with *Proteobacteria*, *Acidobacteria*, and *Actinobacteria* being the predominant groups in untreated soils and in soils treated with organic or inorganic fertilizers.

### Data availability.

Sequences were submitted to the NCBI database in the Sequence Read Archive under accession number SAMN12354609.
